# New Biologics for the Treatment of Atopic Dermatitis: Analysis of Efficacy, Safety, and Paradoxical Atopic Dermatitis Acceleration

**DOI:** 10.1155/2021/5528372

**Published:** 2021-05-30

**Authors:** Hong-jiao Qi, Lin-Feng Li

**Affiliations:** Department of Dermatology, Beijing Friendship Hospital, Capital Medical University, Beijing, China

## Abstract

Atopic dermatitis (AD) is a chronic, inflammatory skin disease with an eczematous rash and itching. Due to undesired adverse effects of traditional systemic treatment, there is still an unmet need for safe and effective long-term therapy for refractory AD. As our understanding of the pathogenesis underlying AD grows, novel treatments targeting specific molecules have been developed. Here, we discuss the efficacy and safety profiles of these drugs in recent clinical trials. Among their adverse effects, of particular note is AD acceleration. Although there is still debate about whether certain adverse reactions can be said to be paradoxical adverse events (PAEs), a wide range of PAEs have been reported during biological treatment for chronic immune-mediated diseases. Close surveillance of novel biologics is crucial to detect new undescribed paradoxical reactions and to shed light on the convoluted pathogenesis of AD.

## 1. Introduction

Atopic dermatitis (AD) is one of the most common chronic, inflammatory, relapsing skin diseases [1]. Up to 17.1% of adults and 22.6% of children are diagnosed with AD each year [2]. AD is a very distressing disease that is characterized by pruritus and dry skin [3]. For patients with moderate-to-severe AD, systematic treatments are often necessary [4]. The use of traditional systemic treatments (systemic corticosteroids, phototherapy, and immunosuppressants) is limited by safety risks and variable therapeutic benefits [5]. Thus, new systemic therapies have been developed recently.

For approximately 20 years, biological agents (BAs) have been widely used in various autoinflammatory and immune diseases [6]. As new emerging drugs come to market, a tradeoff between efficacy and safety is achieved [7]. Dupilumab, an IL-4 and IL-13 inhibitor, was the first biological drug approved by the FDA for the treatment of AD in adults [8]. It is noteworthy for its acceptable low side effect profile (lower rate of conjunctivitis, injection-site reactions, and infections) and high efficacy (36%-44% of patients achieve clear or almost clear skin) [9–11]. Other new biologics that selectively inhibit cytokines involved in the inflammatory component of AD are discussed in our review.

Among the emergent treatment adverse effects, of particular note is AD acceleration. Although there is still a debate about whether certain adverse reactions can be said to be paradoxical adverse events (PAEs), a wide range of PAEs have been reported during biological treatment for chronic immune-mediated diseases [12]. PAEs are defined as the occurrence of a pathological condition that usually responds to this class of drug during biological agent therapy [13].

Insufficient data are available concerning the incidence of PAEs [13]. Most paradoxical reactions have been reported to be connected with anti-TNF therapy; however, it is possible that the number of cases will increase as the number of newly introduced biological agents increases [14]. Representative examples of PAEs are palmoplantar pustular reactions, psoriasiform reactions, and hidradenitis suppurativa (HS) in patients under treatment for rheumatoid arthritis (RA) or inflammatory bowel disease (IBD) [15]. A few reviews and case reports have described PAEs: de novo psoriasis in atopic dermatitis patients treated with dupilumab [16–23], paradoxical head and neck erythema in patients with atopic dermatitis treated with dupilumab [24–26], mepolizumab-induced alopecia in severe eosinophilic asthma [27], and secukinumab-induced exacerbation of previously diagnosed psoriasis [15]. Regarding atopic dermatitis acceleration, two cases reported the exacerbation of atopic dermatitis symptoms by ustekinumab in psoriatic patients [28].

This review discusses the efficacy, safety, and possible PAEs of novel biological therapies currently in phase II and phase III clinical trials for moderate-to-severe AD.

## 2. Pathogenesis

Atopic dermatitis is characterized by T cell-mediated skin inflammation and an impaired skin barrier. The acute phase of AD is characterized by a strong modulation of Th2 and Th22 immune responses, along with effects on the Th17/IL-17 and IL-23 pathways [29, 30]. Barrier-disrupted keratinocytes are potent producers of immunoregulatory cytokines such as thymic stromal lymphopoietin (TSLP), IL-25, and IL-33 [31]. TSLP plays a critical role in activating the Th2 cascade [29]. TSLP and IL-25 activate dendritic cells (DCs) to express OX40 L. OX40 L/OX40 initiates type 2 immune differentiation of T cells. TSLP also induces IL-23 production by human DCs [32]. IL-33 can positively regulate the TSLP-dendritic cell-OX40 L axis, participating in the induction and maintenance of the Th2 response [33, 34]. The Th22 pathway is consistently activated by the Th2 pathway in AD, and both are considered key immune drivers of AD [35, 36]. While acute AD pathogenesis is polarized towards Th2 and Th22 immune responses, chronic AD lesions additionally exhibit a substantial Th1 component. Th2 cells release IL-4, IL-13, IL-31, and IL-5 [31]. IL-4 and IL-13 disrupt barrier function by downregulating filaggrin (FLG) expression. Furthermore, IL-4 and IL-13 prompt inflammation through the stimulation of IgE production from plasma cells and B cell and plasma cell differentiation [37]. IL-4 and IL-13 also amplify IL-31-induced and histamine-induced pruritus [31]. IL-4 and IL-13 augment the production of CCL17, CCL22, and CCL26. These chemokines, along with IL-5, recruit Th2 cells and eosinophils [31, 38]. IL-31 stimulates sensory nerves and induces pruritus [39], the itching evokes scratching, and the itch-scratch cycle aggravates barrier disruption [31].

**Figure 1:**
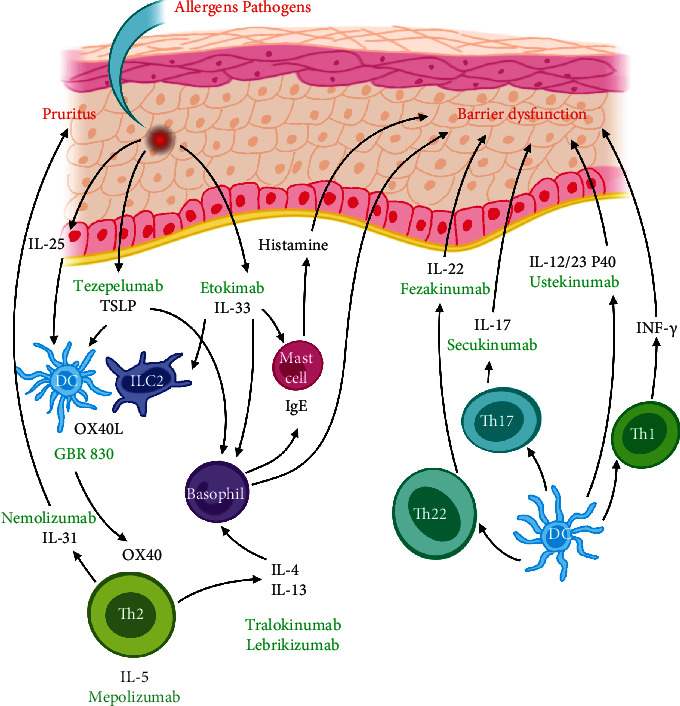
The pathogenesis and new corresponding therapeutic biologics in atopic dermatitis.

IL-22, the leading Th22 cytokine, was suggested to have a major pathogenic role in epidermal pathology, induce keratinocyte proliferation, and downregulate FLG expression, resulting in barrier dysfunction and epidermal hyperplasia [40]. Th17/IL-17 and IL-23 pathway-associated cytokines (IL-17 and IL-12/23p40) are increased in several AD subtypes, including intrinsic [41], Asian [42], and paediatric AD [43]. IL-23 is composed of a p19 subunit in addition to a p40 subunit, which is also a component of IL-12. IL-23 also has one receptor subunit in common with IL-12 and IL-12R*β*1. IL-23 is a crucial player in the expansion and survival of Th17 T cells [44]. IL-17A and IL-17F secreted by Th17 cells can promote eosinophil production [45]. IL-12 secreted by eosinophils, dermal dendritic cells (DDCs), and inflammatory epidermal dendritic cells (IDECs) [45] induces the production of IFN-*γ* [46], which results in the Th2 acute phase-to-Th1 chronic phase switch in AD [45] (Figure 1).

## 3. Efficacy, Safety, and Treatment-Emergent AD Adverse Events

### 3.1. Targeted to IL-33

Etokimab (ANB020) is a humanized anti-human IL-33 monoclonal antibody [47]. A phase 2a clinical trial enrolled 12 moderate-to-severe adult refractory atopic dermatitis patients. A single intravenous dose of placebo (day 7) followed by a single dose of 300 mg intravenous etokimab (day 1) was administered. Eighty-three percent of patients achieved the primary endpoint Eczema Area and Severity Index (EASI) 50, and 33% achieved EASI 75 at day 29. All patients achieved an EASI 50 response on or before day 57. EASI responses were consistent with the improvement of 5D (5-domain) itch scores (5-D pruritus), SCORing Atopic Dermatitis (SCORAD), Dermatology Life Quality Index (DLQI scores), and Investigator Global Assessment (IGA) score 0/1 achievement [47.48]. Etokimab was well tolerated. Most AEs were mild [47]. The most frequent adverse event was dizziness in 17% of the placebo arm versus headache in 25% of the etokimab arm [48].

In a phase 2b randomized, double-blinded, placebo-controlled, multidose study (ATLAS trial), 300 adult moderate-to-severe atopic dermatitis patients were treated with etokimab (ANB020) for 16 weeks (ClinicalTrials.gov Identifier: NCT03533751). Each of the etokimab dosing arms failed to meet the primary endpoint (percent change in EASI from baseline to week 16) (GLOBE NEWSWIRE (https://ir.anaptysbio.com/news-releases/news-release-details/anaptysbio-reports-etokimab-atlas-phase-2b-clinical-trial/) Nov. 08, 2019).

### 3.2. Targeted to OX40

GBR 830 is a humanized mAb (MAB) against OX40, a costimulatory receptor on activated T cells [49]. A phase 2a, randomized, double-blind, placebo-controlled study (NCT02683928) evaluated the safety and biological activity of GBR 830 in adults with moderate-to-severe AD. Sixty-four eligible adult subjects were randomized 3 : 1 to receive 10 mg/kg intravenous GBR 830 or placebo on day 1 and day 29 [49]. Primary endpoints included treatment-emergent adverse events (TEAEs) and changes in epidermal hyperplasia and gene expression of biomarkers in lesional skin biopsies at days 29 and 71 [49]. GBR 830 was well tolerated, with equal TEAE distribution (GBR 830, 63.0% [29/46]; placebo, 63.0% [10/16]). The most frequent TEAEs in the GBR 830 group than in the placebo group were AD, postprocedural infection, and myalgia [49].

Significant reductions in Th1 (IFN-*γ*/CXCL10), Th2 (IL-31/CCL11/CCL17), and Th17/Th22 (IL-23p19/IL-8/S100As) mRNA expression in lesional skin were induced by GBR 830, but the key cytokines Th2 (IL-4 and IL-13) and Th17/Th22 (IL-17A and IL-22) were not significantly reduced with GBR 830 compared with placebo. Hyperplasia measures (thickness/keratin 16/Ki67) were significantly reduced with GBR 830 (*P* < 0.001) [49].

Clinical efficacy was the secondary endpoint. At day 71, the proportion of eligible subjects achieving 50% or greater improvement in EASI score was greater with GBR 830 (76.9% [20/26]) versus placebo (37.5% [3/8]). IGA response (IGA score of 0 or 1) was achieved by 23.1% of GBR 830-treated subjects compared to 12.5% of placebo-treated subjects at day 71. However, SCORAD, body surface area (BSA), and pruritus Numerical Rating Scale (NRS) scores only showed small numerical improvements [49].

### 3.3. Targeted to IL-13

#### 3.3.1. Tralokinumab

Tralokinumab is a fully human monoclonal antibody that potently binds to and neutralizes the effects of IL-13 [50]. In a phase 2b study (NCT02347176), 204 adults were randomized 1 : 1 : 1 : 1 to receive 45, 150, and 300 mg of subcutaneous tralokinumab or placebo every 2 weeks for 12 weeks with concomitant topical glucocorticoids. At week 12, the adjusted mean difference from baseline in EASI score (primary endpoint) was significantly different than that in the placebo group, 150 mg group -4.36 (*P* = 0.03) and 300 mg group -4.94 (*P* = 0.01), while there was no significant difference in the percentage of participants with an IGA response (coprimary endpoint) at week 12 (23.0% vs. 11.8%, *P* = 0.10). More responses were found in participants with greater concentrations of biomarkers (DPP-4 and periostin) [50]. Tralokinumab has an acceptable safety profile with TEAEs relative to placebo (60.8%) and pooled tralokinumab (66%). The most common adverse events were upper respiratory tract infections and headache [50].

In two 52-week, randomized, double-blind, placebo-controlled, phase III trials, ECZTRA 1 and ECZTRA 2 (NCT03131648, NCT03160885), adult patients with moderate-to-severe AD were randomized (3 : 1) to receive subcutaneous tralokinumab 300 mg (600 mg loading dose on day 0) or placebo every 2 weeks (Q2W) for 16 weeks. A total of 802 subjects were enrolled in ECZTRA 1, and 794 subjects were enrolled in ECZTRA 2 [51]. Primary endpoints were IGA score of 0 or 1 and EASI 75 at week 16. Patients achieving an IGA score of 0/1 and/or EASI 75 with tralokinumab at week 16 were rerandomized to tralokinumab 300 mg Q2W, 300 mg Q4W, or placebo for 36 weeks [51]. At week 16, in both trials, the tralokinumab group had a greater proportion of subjects who achieved an IGA score of 0/1 and EASI 75 than placebo. More patients who received tralokinumab vs. placebo achieved an IGA score of 0/1: 15.8% vs. 7.1% in ECZTRA 1 (*P* = 0.002) and 22.2% vs. 10.9% in ECZTRA 2 (*P* < 0.001) and EASI 75: 25.0% vs. 12.7% (*P* < 0.001) and 33.2% vs. 11.4% (*P* < 0.001). More than 50% of patients who achieved IGA 0/1 at week 16 with tralokinumab Q2W maintained that response to week 52 [51]. Tralokinumab has an acceptable safety profile, with a comparable incidence of AEs between tralokinumab and placebo in the initial treatment period of both studies. Among the most often reported AEs, upper respiratory tract infection and conjunctivitis occurred more frequently with tralokinumab than placebo, and atopic dermatitis and skin infection appeared more frequently with placebo [51].

In a phase III, double-blind, randomized, placebo-controlled 32-week trial (ECZTRA 3 NCT03363854), a total of 380 patients were randomized 2 : 1 to receive subcutaneous tralokinumab 300 mg or placebo Q2W with TCS for 16 weeks. Patients who achieved an IGA score of 0/1 and/or EASI 75 at week 16 with tralokinumab were rerandomized 1 : 1 to tralokinumab Q2W or Q4Wfor another 16 weeks [52]. At week 16, more patients treated with tralokinumab than placebo achieved IGA 0/1: 38.9% vs. 26.2% (*P* = 0.015) and EASI 75: 56.0% vs. 35.7% (*P* < 0.001). Of the patients who achieved IGA 0/1 and/or EASI 75 at week 16 with tralokinumab, 89.6% and 92.5% treated with Q2W and 77.6% and 90.8% treated with Q4W maintained a response at week 32, respectively. At week 32, among patients who did not achieve an IGA 0/1 and EASI 75 with tralokinumab Q2W at 16 weeks, 30.5% and 55.8% achieved these endpoints, respectively [52]. The overall rate of adverse events (AEs) was comparable between treatment groups. Viral upper respiratory tract infections, conjunctivitis, headache, upper respiratory tract infections, and injection-site reactions were reported more frequently for tralokinumab than placebo among the most frequently reported AEs [52].

#### 3.3.2. Lebrikizumab

Lebrikizumab is a novel high-affinity monoclonal antibody that binds to soluble IL-13, preventing IL-13R*α*1/IL-4R*α* heterodimerization and inhibiting subsequent signalling [53].

In a phase 2 (NCT02340234) randomized, placebo-controlled, double-blind, phase II study, a total of 209 adult patients with moderate-to-severe AD were randomized (1 : 1 : 1 : 1) to receive a subcutaneous injection of lebrikizumab 125 mg single dose (SD), 250 mg SD, 125 mg Q4W, or placebo Q4W for 12 weeks after a 2-week TCS run-in [54]. At week 12, significantly more patients achieved EASI 50 (primary endpoint) with lebrikizumab 125 mg Q4W (82.4%; *P* = 0.026) versus placebo (62.3%); patients receiving lebrikizumab SD showed no statistically significant improvements in EASI 50 versus placebo. The percentage of patients who achieved IGA 0/1 at week 12 did not show statistical significance in any lebrikizumab group compared with the placebo group [54]. Lebrikizumab was generally well tolerated. The incidence of AEs was similar between groups (66.7% all lebrikizumab vs. 66.0% placebo), mostly mild or moderate. Of the AEs of interest, conjunctivitis (9.6%), herpetic infections (3.8%), and eosinophilia without clinical symptoms (3.2%) occurred more frequently in lebrikizumab-treated patients [54].

In a phase 2b, double-blind, placebo-controlled, randomized clinical trial (NCT03443024), a total of 280 patients were randomized 3 : 3 : 3 : 2 to subcutaneous injections of lebrikizumab at doses of 125 mg every 4 weeks (250 mg loading dose (LD)), 250 mg every 4 weeks (500 mg LD), or 250 mg every 2 weeks (500 mg LD at baseline and week 2) or to placebo every 2 weeks or for 16 weeks [55]. At week 16, the lebrikizumab groups showed significant dose-dependent improvements in the percentage change in EASI score (the primary endpoint) vs. placebo: 125 mg every 4 weeks (-62.3% [37.3%], *P* = 0.02), 250 mg every 4 weeks (-69.2% [38.3%], *P* = 0.002), 250 mg every 2 weeks (­72.1% [37.2%], *P* < 0.001), and placebo (−41.1% [56.5%]). Statistically significantly more patients in the 250 mg lebrikizumab-treated group vs. placebo achieved the following secondary endpoints: IGA 0/1 response, EASI 50, EASI 75, and EASI 90, and pruritus NRS score improvement of ≥4 points at week 16 [55].

Most TEAEs were mild to moderate. TEAEs were reported in 46.2% of placebo patients, 57.5% of 125 mg Q4W patients, 48.8% of 250 mg Q4W patients, and 61.3% of 250 mg Q2W lebrikizumab-treated patients. Among the most frequently reported AEs, upper respiratory tract infections, nasopharyngitis, injection-site pain, and fatigue occurred more frequently with pooled lebrikizumab than with placebo, and headache occurred more frequently with placebo [55].

### 3.4. Targeted to IL-31

Nemolizumab is a humanized monoclonal antibody against the interleukin-31 receptor. In a recently published 16-week, double-blind, phase 3 clinical trial, a total of 215 Japanese patients (≥13 years old) with atopic moderate-to-severe dermatitis were randomized a 2 : 1 ratio to receive subcutaneous nemolizumab 60 mg or placebo every 4 weeks until week 16, with concomitant topical glucocorticoids [56]. At week 16, the mean percent change in the visual analogue scale (VAS) score for pruritus was −42.8% in the nemolizumab group and −21.4% in the placebo group (*P* < 0.001). The mean percent change in the EASI score (one of the secondary outcomes) was −45.9% with nemolizumab and −33.2% with placebo [56].

Nemolizumab was generally well tolerated, with an equal TEAE distribution (nemolizumab, 71%; placebo, 71%). The most commonly reported adverse event of special interest was worsening atopic dermatitis, occurring in 24% of the nemolizumab group and 21% of the placebo group; one patient discontinued nemolizumab as a result. The incidence of injection-related reactions was 8% in the nemolizumab-treated and 3% in the placebo-treated patients [56].

In a randomized, double-blind, phase 2b study (NCT03100344), a total of 226 adult patients with moderate-to-severe AD were randomized 1 : 1 : 1 : 1 to receive subcutaneous injections of nemolizumab 10, 30, and 90 mg every 4 weeks or placebo with topical agents [57]. At week 24, among the three nemolizumab-treated groups, the 30 mg dose had the best response rates. The percentage change in EASI score (the primary endpoint) was statistically significant at the 30 mg nemolizumab dose compared with the placebo (−68.8% vs. −52.1%, *P* = 0.016) and borderline statistically significant at the 10 mg dose (*P* = 0.051). With respect to the secondary endpoints, 36.8% of subjects in the 30 mg nemolizumab group achieved IGA 0/1 versus 21.1% in the placebo group (*P* = 0.06). Compared with the placebo, the 30 mg nemolizumab arm achieved the most distinct improvement in peak pruritus NRS (PP-NRS) scores (−68.6% vs. −34.3%, *P* < 0.0001) [57]. The rate of TEAEs was slightly higher in the nemolizumab groups than in the placebo group. Nonskin infections, including nasopharyngitis, upper respiratory tract infections, and gastroenteritis, occurred more frequently with nemolizumab than with placebo [57].

In a phase 2 (part A) randomized, double-blind, placebo-controlled study (NCT01986933), a total of 264 adult patients with moderate-to-severe AD were randomized 1 : 1 : 1 : 1 to receive subcutaneous nemolizumab (at a dose of 0.1 mg, 0.5 mg, or 2.0 mg per kilogram of body weight) or placebo every 4 weeks or an exploratory dose of 2.0 mg of nemolizumab per kilogram every 8 weeks [58]. At week 12, the percentage changes in the pruritus visual analogue scale (P-VAS) score (the primary endpoint) were −43.7% in the 0.1 mg Q4W group, −59.8% in the 0.5 mg Q4W group, and −63.1% in the 2.0 mg Q4W group versus −20.9% in the placebo group (*P* < 0.01 for all comparisons). For the secondary endpoints, changes in the EASI were −23.0%, −42.3%, and −40.9%, respectively, in the nemolizumab groups versus −26.6% in the placebo group. Changes in BSA were −7.5%, −20.0%, and −19.4%, respectively, in the nemolizumab arms versus −15.7% in the placebo [58]. The incidence of TEAEs was similar among all groups. The most frequent adverse events included exacerbation of AD, nasopharyngitis, upper respiratory tract infection, peripheral oedema, and increased creatine kinase levels. Exacerbation of AD and peripheral oedema were more common in the nemolizumab groups than in the placebo group [58].

In a 52-week double-blind extension phase II (part B) trial (NCT01986933), long-term efficacy and safety were assessed in patients who completed part A of the study. Previous placebo patients in part A were rerandomized 1 : 1 : 1 to receive subcutaneous nemolizumab (0.1, 0.5, or 2.0 mg/kg Q4W) in part B [59]. The improvement from baseline on the pruritus visual analogue scale (VAS) score was maintained or increased from weeks 12 to 64. The greatest improvement was observed in the 0.5 mg/kg nemolizumab group [59].

At week 64, percentage improvements from baseline in VAS score were −73.0%, −89.6%, −74.7%, and −79.1% in the 0.1, 0.5, and 2.0 mg/kg Q4W and 2.0 mg/kg Q8W groups, respectively. Changes from baseline in EASI score were −68.5%, −75.8%, −78.9%, and −69.3% in the 0.1, 0.5, and 2.0 mg/kg Q4W and 2.0 mg/kg Q8W groups, respectively [59]. No new safety concerns were observed after long-term use of nemolizumab. Exacerbation of AD (8%), upper respiratory tract infection (4%), nasopharyngitis (4%), peripheral oedema (3%), increased blood creatine phosphokinase level (3%), and injection-site reaction (2%) were the most common treatment-related AEs in the study [59].

Among the abovementioned trials, exacerbation of AD occurred more frequently in the placebo group in the phase 2B study [57], whereas it occurred more often in the nemolizumab groups in the phase 3 study [56] and phase 2 part A [58]+part B study [59].

### 3.5. Targeted to IL-22

Fezakinumab (ILV-094) is a human monoclonal antibody that directly binds to IL-22 [60]. In a phase 2a randomized, double-blind, placebo-controlled trial (NCT01941537), a total of 60 adult patients were randomized (2 : 1) to receive intravenous 300 mg fezakinumab (loading dose of 600 mg at baseline) or placebo every 2 wks for 10 wks [60]. At week 12, the mean decline in SCORAD (primary endpoint) for the entire study population was 13.8 ± 2.7 (fezakinumab) and 8.0 ± 3.1 (placebo), *P* = 0.134. In the severe AD patient subgroup (baseline SCORAD ≥ 50), the SCORAD reduction was significantly larger in the fezakinumab group than in the placebo group (21.6 ± 3.8 vs. 9.6 ± 4.2, *P* = 0.029) and at 20 weeks (27.4 ± 3.9 vs. 11.5 ± 5.1, *P* = 0.010). In nonsevere (moderate) AD patients, none of the efficacy endpoints showed statistically significant differences between the fezakinumab and placebo arms [60]. Adverse events occurred at similar rates between the fezakinumab and placebo groups. Among the most common adverse events that occurred more frequently in the fezakinumab group vs. the placebo group were viral upper respiratory tract infections, occurring in 4 patients receiving fezakinumab [60].

### 3.6. Targeted to IL-5, IL-17A, TSLP, and IL-12/23p40

Mepolizumab (SB240563): anti-IL-5 [61, 62], secukinumab: anti-IL-17A [63], tezepelumab (AMG 157/MEDI9929): anti-TSLP [64], and ustekinumab: anti-IL12/23p40 [65, 66] all failed to reach their primary endpoint (Table 1).

## 4. Discussion

Among the novel agents discussed here, GBR 830, tralokinumab, lebrikizumab, and nemolizumab have different extents of efficacy, and etokimab showed inconsistent results from different trials. Fezakinumab (targeted to IL-22) resulted in significant improvements for only severe patients (SCORAD ≥ 50) [60]. Other drugs, such as tezepelumab (targeted to TSLP), mepolizumab (targeted to IL-5), secukinumab (targeted to IL-17A), and ustekinumab (targeted to IL12/23p40), failed to reach their primary endpoint in clinical trials (Tables 2 and 1).

Among these aforementioned agents, some drugs showed more frequent atopic dermatitis TEAEs than the placebo, including nemolizumab and GBR 830 (Table 3). This atopic dermatitis as a safety outcome was not associated with the efficacy outcome, opening the question of whether some of these cases are paradoxical adverse events. According to the data we showed above and the definition of PAE, some of PAEs may have occurred with these drugs, especially nemolizumab and maybe GBR 830 and the others.

Mechanisms involved in PAEs are complicated. Limited hypotheses have been proposed based on TNF inhibitor investigations. First, an imbalance in the cytokine milieu is advanced during most PAEs [13]. The prototypical example is biological agent-induced psoriasis, due to a TNF-*α*/type-1 IFN cytokine imbalance: TNF-inhibitors (TNFi) block TNF-*α*, which results in uncontrolled activation of plasmacytoid dendritic cells (pDCs), with surplus production of IFN-*α*. IFN-*α* drives paradoxical skin inflammation [67]. Atopic dermatitis is also an autoimmune disease that involves several cytokines. These cytokines are interwoven in the pathogenesis of AD, and targeting one of these cytokines may have effects on the others. Second, individual genetic susceptibility might play a role [68]. The relationship between AD and single nucleotide polymorphisms of some genes has been investigated, such as SNPs of the interleukin-4/interleukin-13 receptor gene and the *β*-defensin 1 gene [69, 70]. These polymorphisms influence the genes involved in cytokine production, and it is probable that paradoxical reactions occur in patients with an underlying genetic predisposition [14]. This might be one reason why biological agents have been used successfully in some patients with atopic dermatitis, while paradoxically these same types of atopic dermatitis are triggered by the same biological agents. Third, there is a shift in the cutaneous immune response pattern, for example, psoriatic morphology changes (plaque to pustular) [67]. Fourth, a spatial shift of immune cells to the skin [67], for example, a spatial shift of lymphocytes from the gastrointestinal system to the skin, gives rise to the development of psoriasis-like skin inflammation in patients treated for IBD [71]. Fifth, imbalance or dysfunction of regulatory T cells [67], paradoxical cutaneous sarcoidosis and granulomatous disease are prime examples. Drugs targeting TNF result in TNF-*α*/IL-10 cytokine imbalance and a decrease in TNFR2, followed by dysfunctional Treg increases [72].

Although a few AD cases of paradoxical reactions to biological therapy have been reported, with their increasing use for AD, an increasing number of reports of paradoxical adverse events of AD might be seen. Recently, a systematic review of paradoxical eruptions in response to targeted therapies in dermatology was published, and they identified that TNF-*α* inhibitors resulted in 91.2% (1869/2049) of all cases, followed by IL-17/17R (3.5%), IL-4R*α* (2.7%), IL 12/23 (2.4%), and IL-23 (0.01%) inhibitors in 2049 cases of paradoxical reactions. Psoriasiform and eczematous eruptions were the most commonly reported [73].

Biological therapies associated with PAE onset are a challenging issue. Therefore, careful clinical and immunological evaluation should accompany the initiation of biological therapies. In addition, closely monitoring patients receiving biological treatment to detect such reactions is also recommended. These countermeasures will extend our clinical knowledge and shed light on our understanding of the complex immune mechanisms underlying PAEs. The understanding of these new types of adverse reactions will help us to optimize our choices for atopic dermatitis treatment.

## Figures and Tables

**Table 1:** Summary of the new biologics for atopic dermatitis in clinical trials (drugs that did not achieve the primary endpoint^∗^).

Drug Mechanism f action	Phase trial ClinicalTrials.gov identifier	*N* (ratio), age	Duration	TCS (Y/N/R)	Primary endpoint	% achieving primary endpoint
Fezakinumab (ILV-094) Anti-IL-22	Phase IIa [60] NCT01941537	60 (2 : 1), adults	20 wks	N	SCORAD	Entire population: 300 mg iv Q2W: 13.8 ± 2.7 vs. placebo: 8.0 ± 3.1, *P* = 0.134 SCORAD ≥ 50 subgroup: 300 mg: 21.6 ± 3.8 vs. placebo: 9.6 ± 4.2, *P* = 0.029

Mepolizumab (SB240563) Anti-IL-5	Phase II [61] N/A	34 (1 : 1), adults	20 wks	N	IGA 0/1 plus ≥ 2-point (week 16)	Failed (100 mg SC Q4W)
	N/A [62]	43, adults	30 days	R	PGA (14 days)	Failed: two single doses of 750 mg iv, *P* = 0.115

Secukinumab Anti-IL-17A	Phase II [63] NCT02594098	41 (2 : 1), adults	16 wks	N	Reduction of epidermal thickness EASI (week 16)	Failed: 300 mg qw through week 4, followed by Q4W to week 16

Tezepelumab (AMG 157/MEDI9929) Anti-TSLP	Phase IIa [64] NCT02525094	113 (1 : 1), adults	24 wks	Y	EASI 50 (week 12)	Failed: 280 mg SC Q2W: 64.7% vs. placebo: 48.2%, *P* = 0.091

Ustekinumab Anti-IL12/23p40	Phase II [65] NCT01806662	33 (1 : 1), adults	32 wks	Y	SCORAD 50 (week 16)	Failed (45 mg or 90 mg SC 3 doses)
	Phase II [66] N/A	79 (1 : 1 : 1) (Japanese), adults	24 wks	Y	EASI score (week 12)	Failed (45 mg or 90 mg SC 2 doses)

PGA: physician's global assessment. ^∗^Fezakinumab (ILV-094): the SCORAD ≥ 50 subgroup achieved the primary endpoint.

**Table 2:** Summary of the new biologics (drugs achieving the primary endpoint) for AD and paradoxical AD acceleration (drug < placebo) in clinical trials.

Drug Mechanism of action	Phase trial ClinicalTrials.gov identifier	*N* (ratio), age, duration TCS (yes/no/rescue)	Primary endpoint	% achieving primary endpoint	Parameters related to pruritus instead	TEAE	Interested TEAEs: atopic dermatitis
Etokimab (ANB020) Anti-IL-33	Phase IIa [47, 48] N/A	12 (N/A), adults, 140 days R	EASI 50 (day 29)	83%	5-D pruritus: *P* < 0.001	Headache	One patient had secondarily infected AD
	Phase IIb (ATLAS) NCT03533751	300 (N/A), adults, 16 wks N/A	EASI (week 16)	Failed	N/A	N/A	N/A

Tralokinumab Anti-IL-13	Phase IIb [50] NCT02347176	204 (1 : 1 : 1 : 1), adults, 12 wks Y	EASI (week 12)	(Adjusted mean difference) 150 mg Q2W: -4.36, *P* = 0.03 300 mg Q2W: -4.94, *P* = 0.01	PP-NRS scores (week 12) 45 mg: -0.77, *P* = 0.04 300 mg: -1.14, *P* = 0.002	Most frequent TEAEs: URTI and headache Pooled tralokinumab > placebo: nasopharyngitis, headache, and injection-site reaction	Placebo: 4 (7.8) Pooled tralokinumab: 9 (5.9) 45 mg: 3 (6.0) 150 mg: 3 (5.9) 300 mg: 3 (5.8)
	Phase III [51] ECZTRA 1 NCT03131648	802 (3 : 1), adults, 52 wks R	IGA 0 or 1 EASI 75 (week 16)	IGA: 300 mg SC Q2W: 15.8% vs. placebo: 7.1%, *P* = 0.002 EASI 75: 300 mg: 25.0% vs. placebo: 12.7%, *P* < 0.001	PP-NRS ≥ 4-point (week 16) 300 mg Q2W: 20.0% vs. placebo: 10.3%, *P* = 0.002	Tralokinumab > placebo: URTI, conjunctivitis Placebo > Tralokinumab: atopic dermatitis, skin infection	Placebo: 75 (38·3) Tralokinumab: 156 (25·9)
	Phase III [51] ECZTRA 2 NCT03160885	794 (3 : 1), adults, 52 wks R	IGA 0 or 1 EASI 75 (week 16)	IGA: 300 mg SC Q2W: 22.2% vs. placebo: 10.9%, *P* < 0.001 EASI 75: 300 mg: 33.2% vs. placebo: 11.4%, *P* < 0.001	PP-NRS ≥ 4-point (week 16) 300 mg Q2W: 25.0% vs. placebo: 9.5%, *P* < 0.001	Tralokinumab > placebo: URTI, conjunctivitis Placebo > tralokinumab: atopic dermatitis, skin infection	Placebo: 67 (33.5) Tralokinumab: 98 (16.6)
	Phase III [52] ECZTRA 3 NCT03363854	380 (2 : 1), adults, 32 wks Y	IGA 0 or 1 EASI 75 (week 16)	IGA: 300 mg SC Q2W: 38.9% vs. placebo: 26.2%, *P* = 0.015 EASI 75: 300 mg: 56.0% vs. placebo: 35.7%, *P* < 0.001	PP-NRS ≥ 4-point (week 16) 300 mg: 45.4% vs. placebo: 34.1%, *P* = 0.037	Viral upper respiratory tract infection, conjunctivitis, headache, URTI, and injection-site reaction	Placebo: 10 (7.9) Tralokinumab: 6 (2.4)

Lebrikizumab Anti-IL-13	Phase II [54] TREBLE NCT02340234	209 (1 : 1 : 1 : 1), adults, 12 wks Y	EASI 50 (week 12)	125 mg SC Q4W: 82.4% vs. placebo 62.3%, *P* = 0.026; 125 mg SC and 250 mg SC SD did not meet	Pruritus VAS (125 mg SD: -34.9%, 250 mg SD: -32.8%, 125 mg Q4W: -40.7% vs. placebo: -27.5%) was not statistically significant in all the groups	Conjunctivitis, herpetic infections, and eosinophilia occurred more often in lebrikizumab	N/A
	Phase IIb [55] NCT03443024	280 (3 : 3 : 3 : 2), adults, 16 wks	EASI (week 16)	125 mg SC Q4W: -62.3%, *P* = 0.02 250 mg SC Q4W: -69.2%, *P* = 0.002 250 mg SC Q2W: -72.1% (*P* < 0.001) vs. placebo: -41.1%	PP-NRS ≥ 4-point (week 16) 125 mg Q4W: 41.8%, *P* = 0.24 250 mg Q4W: 47.4%, *P* = 0.11 250 mg Q2W: 70.0% (*P* < 0.001) vs. placebo: 27.3%	Pooled lebrikizumab > placebo: UPTI, nasopharyngitis, injection-site pain, and fatigue	N/A

URTI: upper respiratory tract infection.

**Table 3:** Summary of the new biologics (drugs achieved their primary endpoint) for AD and paradoxical AD acceleration (drug > placebo) in clinical trials.

Drug Mechanism of action	Phase trial ClinicalTrials.gov identifier	*N* (ratio), age, duration TCS (Y/N/R)	Primary endpoint	% achieving primary endpoint	Parameters related to pruritus	TEAE	Interested TEAEs: atopic dermatitis
Nemolizumab Anti-IL-31	Phase III [56] N/A	215 (2 : 1), Japanese, age ≥ 13, 16 wks Y	VAS for pruritus (week 16)	60 mg SC Q4W: -42.8% vs. placebo: -21.4%, *P* < 0.001	N/A	Worsening of AD and atopic dermatitis, injection-related reaction, increased TARC level, increased blood CK were more common in the nemolizumab	Worsening of AD Nemolizumab: 34 (24) Placebo: 15 (21) Atopic dermatitis Nemolizumab: 33 (23) Placebo: 15 (21)
	Phase IIb NCT03100344 [57]	226 (1 : 1 : 1 : 1), adults, 24 wks Y	EASI (week 24)	30 mg SC Q4W: -68.8% vs. placebo: -52.1%, *P* = 0.016 10 mg SC Q4W and 90 mg SC Q4W failed to meet primary endpoint	PP-NRS ≥ 4-point (week 24) 30 mg Q4W: 17.5% vs. placebo: 5.3%, *P* ≤ 0.01 10 mg Q4W vs. placebo: *P* ≤ 0.05 90 mg Q4W vs. placebo: *P* ≤ 0.05	Nasopharyngitis, URTI, and gastroenteritis were more common in the nemolizumab	AD exacerbation Placebo: 9 (16.1%) 10 mg: 3 (5.5%) 30 mg: 7 (12.3%) 90 mg: 7 (12.3%) All nemolizumab: 17 (10.1%) Atopic dermatitis Placebo: 18 (32.1%) 10 mg: 12 (21.8%) 30 mg: 14 (24.6%) 90 mg: 16 (28.1%) All nemolizumab: 42 (24.9%)
	Phase II NCT01986933 part A [58]	264 (1 : 1 : 1 : 1 : 1), adults, 12 wks R	VAS for pruritus (week 12)	0.1 mg/kg SC Q4W: -43.7%, *P* = 0.002 0.5 mg/kg SC Q4W: -59.8%, *P* < 0.001 2.0 mg/kg SC Q4W: -63.1%, (*P* < 0.001) vs. placebo: -20.9%	N/A	Exacerbation of AD and peripheral oedema were more common in the nemolizumab groups	3 SAE of exacerbation of AD 10 patients who discontinued treatment related to AD all in exacerbation of AD Placebo: 7 (13) Nemolizumab 0.1 mg/kg: 11 (21) 0.5 mg/kg: 10 (19) 2.0 mg/kg Q4W: 11 (21) 2.0 mg/kg Q8W: 9 (17)
	Phase II NCT01986933 part B [59]	191 (1 : 1 : 1 : 1 : 1), adults, 52 wks R	VAS for pruritus (week 12) was assessed during part A	N/A	Pruritus VAS score (week 64): the improvement in part A was maintained or increased from week 12 to week 64	Exacerbation of AD, URTI, nasopharyngitis, peripheral oedema, increased blood CPK level, and injection-site reaction were the most common treatment-related AEs	Exacerbation of AD (64-week study period) 0.1 mg/kg: 15 (28) 0.5 mg/kg: 13 (24) 2.0 mg/kg Q4W: 14 (27) 2.0 mg/kg Q8W: 11 (21)

GBR 830 Anti-OX40	Phase IIa [49] NCT02683928	64 (3 : 1), adults, 85 days N	TEAEs and changes in epidermal hyperplasia/cytokines (days 29 and 71)	Equal TEAE (63.0% vs. 63.0%) Significant reductions in hyperplasia, OX40+ T OX40 L+ dendritic cells mRNA of TH1, TH2, and TH17/TH22	N/A	AD, postprocedural infection, and myalgia were more common in GBR 830	Placebo: 2 (12.5) GBR 830: 6 (13.0)

TARC: thymus and activation-regulated chemokine; CK/CPK: creatine kinase/creatine phosphokinase (CPK).
